# The health consequences of child marriage: a systematic review of the evidence

**DOI:** 10.1186/s12889-022-12707-x

**Published:** 2022-02-14

**Authors:** Suiqiong Fan, Alissa Koski

**Affiliations:** 1 Department of Epidemiology, Biostatistics and Occupational Health, McGill University, 2001 McGill College Avenue, Montreal, Quebec H3A 1G1 Canada; 2 Institute for Health and Social Policy, McGill University, 2001 McGill College Avenue, Montreal, Quebec H3A 1G1 Canada

**Keywords:** Child marriage, Social determinants of health, Systematic review

## Abstract

**Background:**

Child marriage, defined as marriage before 18 years of age, is a violation of human rights and a marker of gender inequality. Growing attention to this issue on the global development agenda also reflects concerns that it may negatively impact health. We conducted a systematic review to synthesize existing research on the consequences of child marriage on health and to assess the risk of bias in this body of literature.

**Methods and findings:**

We searched databases focused on biomedicine and global health for studies that estimated the effect of marrying before the age of 18 on any physical or mental health outcome or health behaviour. We identified 58 eligible articles, nearly all of which relied on cross-sectional data sources from sub-Saharan Africa or South Asia. The most studied health outcomes were indicators of fertility and fertility control, maternal health care, and intimate partner violence. All studies were at serious to critical risk of bias. Research consistently found that women who marry before the age of 18 begin having children at earlier ages and give birth to a larger number of children when compared to those who marry at 18 or later, but whether these outcomes were desired was not considered. Across studies, women who married as children were also consistently less likely to give birth in health care facilities or with assistance from skilled providers. Studies also uniformly concluded that child marriage increases the likelihood of experiencing physical violence from an intimate partner. However, research in many other domains, including use of contraception, unwanted pregnancy, and sexual violence came to divergent conclusions and challenge some common narratives regarding child marriage.

**Conclusions:**

There are many reasons to be concerned about child marriage. However, evidence that child marriage causes the health outcomes described in this review is severely limited. There is more heterogeneity in the results of these studies than is often recognized. For these reasons, greater caution is warranted when discussing the potential impact of child marriage on health. We provide suggestions for avoiding common biases and improving the strength of the evidence on this subject.

**Trial registration:**

The protocol of this systematic review was registered with PROSPERO (CRD42020182652) in May 2020.

**Supplementary Information:**

The online version contains supplementary material available at 10.1186/s12889-022-12707-x.

## Introduction

Marriage before the age of 18, often referred to as child marriage, is a violation of human rights that hinders educational attainment and literacy and may increase the likelihood of living in poverty in adulthood [1–5]. Girls are far more likely to marry than boys, and these consequences contribute to existing gender gaps in educational outcomes in some settings [6, 7]. The United Nations Sustainable Development Goals list child marriage as an indicator of gender inequality and call for an end to the practice by the year 2030 [8]. Child marriage remains ongoing throughout much of the world despite intensifying efforts to eliminate it [9].

In addition to its consequences on education, growing attention to child marriage as a global development issue also seems to reflect increasing consideration of its potential impacts on population health. Multinational organizations including the World Bank, the United Nations Population Fund (UNFPA), and the United Nations Children’s Fund (UNICEF) include the potential for harmful consequences on health among the foremost concerns regarding this practice [2, 10–13]. These organizations highlight relationships between child marriage and early childbearing [11–13], obstetric complications [12, 13], violence [2, 12], and sexually transmitted infections [12], among other adverse outcomes.

We undertook this systematic review to synthesize the results of existing research regarding the impact of child marriage on the health of persons who marry before the age of 18. We evaluated the range of health outcomes that have been studied and the geographic distribution of those studies. We also assessed the risk of bias in individual studies and the likelihood that their results reflect causal relationships.

## Methods

We searched three databases for literature on the relationship between child marriage and health: MEDLINE, Embase, and Ovid Global Health. These databases were chosen because they focus on biomedicine and human health. We aimed to include as broad a range of health outcomes as possible and focusing our search within these databases allowed us to avoid defining specific health outcomes within our search terms. Instead, we searched for studies of child marriage within these databases. This approach made our search terms more concise and the range of outcomes more inclusive. Specific search terms used for each database are included in Supplementary File 1. We registered our protocol with PROSPERO (CRD42020182652) in May 2020 and conducted our database searches shortly afterward.

We also searched Google Scholar to identify relevant grey literature. Haddaway et al. [14] found that the majority of grey literature tends to appear within the first 200 citations returned by Google Scholar and recommend focusing on the first 200-300 records. We followed this recommendation and evaluated the first 300 records returned, as sorted by relevance. Search terms used in Google Scholar are also included in Supplementary File 1. We reviewed the bibliographies of all included studies in an effort to identify any relevant citations not picked up through searches of the databases described above. The search strategy was developed with assistance from a research librarian at McGill University.

Citations returned from searches of all four databases were imported into EndNote X9 and duplicate citations removed [15]. We transferred all unique citations into Rayyan to facilitate the review process [16]. A single reviewer (SF) examined the title and abstract of each unique citation for eligibility according to pre-defined criteria specified in the registered protocol. Articles were brought forward for full-text review if they described etiologic studies that used quantitative methods to estimate the effect of child marriage on one or more health outcomes. We defined child marriage as formal or informal union prior to the age of 18. If the title and abstract did not specify the age thresholds used to define child marriage, they were brought forward for full-text review. For example, abstracts that referred to the effect of adolescent or teen marriage without explicitly stating how those exposures were defined were brought forward. Eligible health outcomes included physical or mental health disorders or symptoms of those disorders, as well as health behaviours. Eligible health behaviours included actions like smoking or dietary habits as well as health care seeking, such as prenatal care. We restricted our review to studies in which outcomes were measured at the individual level and to those that measured the effect of child marriage on the individuals married; studies that examined the effect of age at marriage on the offspring of the persons who married were excluded. Studies written in English, French or Chinese were eligible for inclusion.

We excluded studies that used solely qualitative methods and quantitative studies that relied exclusively on hypothesis testing to indicate differences between groups. For example, studies that used chi-squared tests to indicate whether the distribution of some characteristic differed between persons married before the age of 18 and those married at older ages were excluded, even if the authors seemed to interpret their results as causal, because such testing does not result in a comparative effect measure (e.g., a risk difference or an odds ratio) and does not account for potential biases. We also excluded studies in which persons who married before the age of 18 were incorporated into a larger aggregate age category, making the effect of child marriage unidentifiable. For example, comparisons of outcomes among persons who married between 15 and 19 years of age with those who married between 20 and 24 years of age were not eligible for inclusion. Conference presentations and abstracts were also excluded.

Both authors read the full text of each article brought forward from the title and abstract review and independently judged their eligibility according to the inclusion and exclusion criteria described above. Discrepancies were resolved through discussion. The following information was extracted from each included study: authors, title, year of publication, the language of publication, country/region in which the study was conducted, study design, study population, sample size, data sources, statistical methods, outcomes, and results.

### Risk of bias assessment

We assessed the risk of bias within each included study using the Risk Of Bias In Non-randomised Studies - of Interventions (ROBINS-I) tool developed by members of the Cochrane Bias Methods Group and the Cochrane Non-Randomised Studies Methods Group [17]. ROBINS-I is designed to evaluate the risk of bias in non-randomized studies by considering how closely the study’s design and methods approximate an ideal randomized trial. To illustrate, in a hypothetical cluster-randomized trial to estimate the causal effect of child marriage on a specified health outcome, the treatment or intervention would be marriage before the age of 18 years. All children in a specific area (a region, a state, a community, etc.) would be randomized at a very young age to one of two treatment groups: those randomized to the intervention would marry at some point prior to their 18th birthdays (a = 1), while those randomized to the control group would marry on their 18th birthday or any later age (a = 0). All children would then be followed up over a period of time sufficient to observe the specified outcome of interest. In the ideal randomized trial, all persons would adhere to their assigned treatment (i.e., remain married) and would remain in the study until follow-up was complete. After the follow-up period, the probability of the outcome among those assigned to a = 1 would be compared with the same probability among those assigned to a = 0. Under these conditions, we could expect that there would be no differences between those children who married before the age of 18 and those who married afterward aside from age at marriage. As a result, if the probability of the outcome among those randomly assigned to marry as children differed from the probability among those randomly assigned to marry after their 18th birthdays, one could interpret that difference as the causal effect of child marriage [18].

Of course, a randomized trial like this would be unethical and could never actually be conducted. Researchers interested in the effects of child marriage on health must rely on non-randomized study designs to estimate the causal effect of interest. Without the benefit of randomization, it becomes challenging to identify the causal effect of child marriage because those who marry as children are different from those who marry at later ages in many ways. For example, girls who marry before the age of 18 come from poorer households and from communities with greater gender inequality, on average, compared to those who marry at later ages. These differences are likely to affect their health through causal pathways other than age at marriage, such as the experience of violence or limited ability to access education or health care. This means that a naïve comparison of health outcomes between those who marry as children and those who marry as adults is likely to mix up the consequences of age at marriage with the consequences of childhood poverty and gender inequality.

The ROBINS-I tool requires assessors to carefully consider the potential for multiple sources of bias including confounding, inappropriate selection of participants into the study (i.e., selection bias), mishandling of missing data, and problems with the measurement of exposures and outcomes (i.e., information bias). The potential for bias in each domain is assessed through a series of signaling questions and a summary judgement of low, moderate, serious, or critical risk of bias is then made within each domain. A cross-domain judgement of the risk of bias for the entire study is made based on the risk within each individual domain. Both authors independently assessed the risk of bias in each included study. Disagreements in any single domain or across domains were resolved by discussion.

**Fig. 1:**
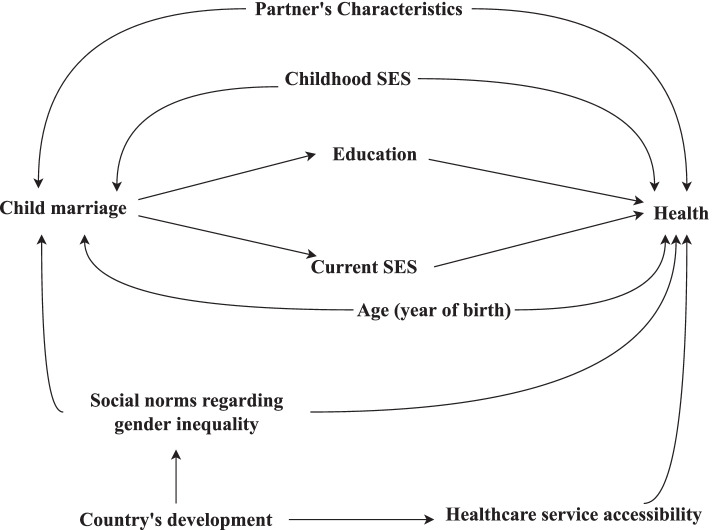
Directed acyclic graph illustrating assumed causal relationships between child marriage and a wide range of health outcomes

We identified a set of variables likely to confound estimates of the effect of child marriage on a wide range of health outcomes in advance to facilitate assessment of bias in this domain. These variables and their relationships to child marriage and health, broadly defined, are illustrated in the simplified Directed Acyclic Graph (DAG) in Fig. 1. The prevalence of child marriage has fallen over time in many countries, which means that the likelihood of marrying before the age of 18 differs across birth cohorts [6, 19]. As discussed above, childhood socioeconomic conditions and gender inequality may lead to child marriage. They may also influence health later in life through a variety of causal pathways. We also considered spousal characteristics a source of confounding because the presence of an available spouse may drive child marriage. For example, a potential husband willing to pay a bride price for a young wife may motivate a family to marry a girl child. The same characteristics of the spouse that may motivate the marriage, such as his age, wealth, and attitudes regarding gender equity, may influence the married child’s health later in life through mechanisms like controlling behaviour. In studies that use pooled data from across multiple regions or countries, it is also important to control for confounding by country/regional-level variables that affect both the probability of child marriage and health. The DAG also illustrates our assumption that the effects of child marriage on health are often mediated through educational attainment and socioeconomic conditions after marriage.

### Analysis

We synthesized results narratively. Included studies considered a wide range of health outcomes, as intended given our search strategy. We found it most intuitive and pragmatic to synthesize results within broad outcome categories, such as the effects of child marriage on contraceptive use, on maternal health care, and on mental health. These categories emerged from the data and were not pre-specified. Meta-analyses were not conducted because the studies examined a wide range of health outcomes that were measured in different ways. The serious risk of bias in all included studies, discussed below, also made quantitative synthesis inappropriate.

## Results

**Fig. 2:**
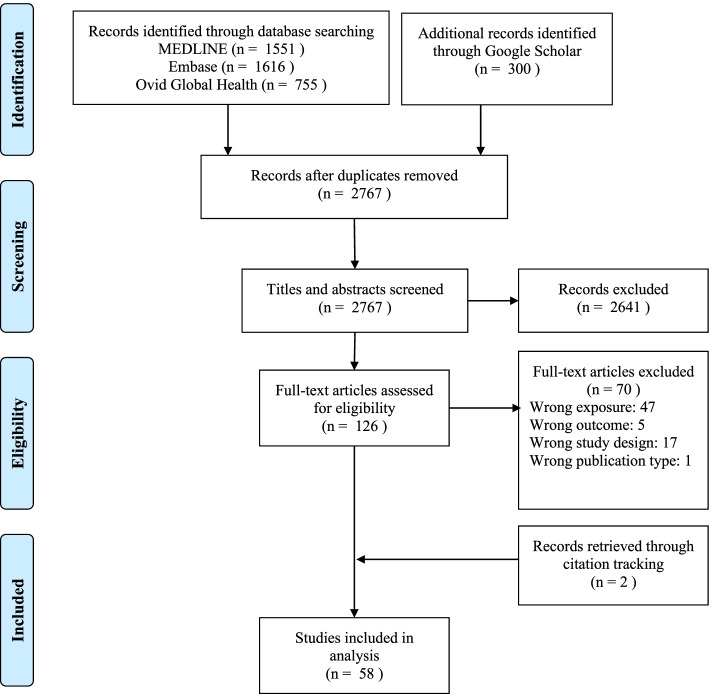
PRISMA flow diagram illustrating the process used to identify eligible studies

Our search strategy returned a total of 2767 unique records from MEDLINE, Embase, Ovid Global Health and Google Scholar, as shown in Fig. 2. After title and abstracting screening, the full text of 126 articles was reviewed. Fifty-six of these studies met our inclusion criteria and two additional eligible studies were identified through citation tracking, for a total of 58 included articles.

**Fig. 3:**
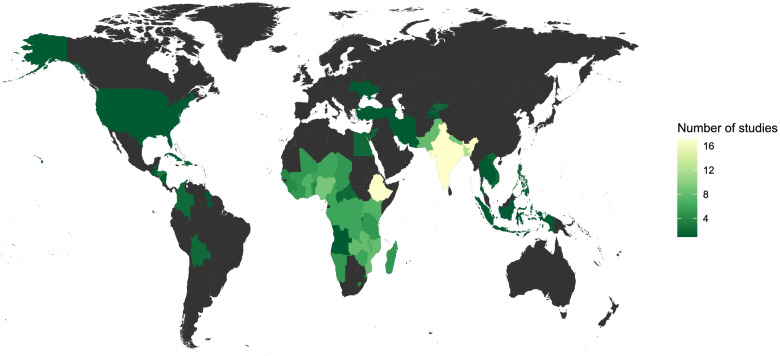
Geographic distribution of included studies

Selected characteristics of all 58 studies included in our review are presented in Table 1. These studies were published between 1989 and 2020 but the vast majority (*n* = 55, 95%) were published in 2010 or later and more than half (*n* = 31, 53%) were published between 2016 and 2020, which reflects the relatively recent rise of child marriage on global health and development agendas. Included studies were based in 70 countries across the globe, as illustrated in Fig. 3. Nearly all studies, 57 of 58, were based in low- and middle-income countries according to World Bank classifications [20]; the single exception was a study based in the United States [21]. The geographic distribution of studies included in our review was heavily focused in South Asia (*n* = 30, 52%) and Sub-Saharan Africa (*n* = 27, 47%), which is perhaps unsurprising given that countries in these regions have some of the highest rates of child marriage in the world [9]. However, more than half of the studies included in our review were based in just three countries: India (*n* = 13), Bangladesh (*n* = 8) and Ethiopia (*n* = 11). Studies from regions other than South Asia or Sub-Saharan Africa were nearly all included in a handful of studies that analyzed survey data from multiple countries simultaneously [22–24].

Nearly all included studies, 55 of 58 (95%), were based on the analysis of cross-sectional survey data. More than half (*n* = 34, 59%) relied on data from a single source, the Demographic and Health Surveys (DHS), or their precursor, the World Fertility Surveys (WFS).

**Table 1:** Selected characteristics of studies included in the systematic review

Authors	Year	Country	Design	Sample size	Outcomes	Risk of Bias
Agyei and Mbamanya [25]	1989	Kenya	CS (WFS)	6193	Number and timing of births	Serious
Ali et al. [26]	2020	Bangladesh	CS (DHS)	15842	Number and timing of births	Serious
Ayane et al. [27]	2019	Ethiopia	CS	314	Birth intervals	Serious
Baytekus et al. [28]	2019	Ethiopia	CS	742	Nutritional status	Serious
Begum et al. [29]	2015	India	CS	1137	Intimate partner violence	Serious
Berlie and Alamerew [30]	2018	Ethiopia	CS	201	Number and timing of births	Serious
Birhanu et al. [31]	2019	Ethiopia	CS (DHS)	2679	Number and timing of births	Critical
De Groot et al. [32]	2018	Ghana	CS	2497	Number and timing of births; contraception; mental health; others	Serious-Critical^a^
Delprato and Akyeampong [33]	2017	39 countries in Sub-Saharan Africa and Southwest Asia	CS (DHS)	from 21544 to 209617	Maternal health care	Serious
Efevbera et al. [34]	2019	35 countries in Sub-Saharan Africa	CS (DHS)	249269	Nutritional status; number and timing of births	Serious
Erulkar [35]	2013	Ethiopia	CS	1506	Intimate partner violence	Serious
Fakhari et al. [36]	2020	Iran	CS	530	Mental health	Serious
Gebremedhin and Betre [37]	2009	Ethiopia	CS	1376	Number and timing of birth	Serious
Gebrezgi et al. [38]	2017	Ethiopia	CS	422	Intimate partner violence	Serious
Godha et al. [39]	2013	India, Bangladesh, Nepal, Pakistan	CS (DHS)	14628 (India), 2129 (Bangladesh), 1658(Nepal), 1546 (Pakistan)	Number of timing of births; birth intervals; unwanted or mistimed pregnancy, and pregnancy termination; contraception; maternal health care	Serious
Habyarimana and Ramroop [40]	2018	Rwanda	CS (DHS)	6841	Contraceptive use	Serious
Hailemariam and Haddis [41]	2011	Ethiopia	CS (DHS)	4121	Contraceptive use	Serious
Hong Le et al. [42]	2014	Viet Nam	CS	1701	Intimate partner violence	Serious
Imasiku et al. [43]	2013	Zambia	CS (DHS)	4343	Contraceptive use	Serious
John, Edmeades, and Murithi [44]	2019	Niger and Ethiopia	CS	2463 (Niger), 3501 (Ethiopia)	Mental health	Serious
John, Edmeades, Murithi, et al. [45]	2019	Ethiopia	CS	3396	Mental health	Serious
Kamal [46]	2012	Bangladesh	CS (DHS)	9572	Number of timing of births; unwanted or mistimed pregnancy, and pregnancy termination; contraceptive use	Serious
Kamal [47]	2013	Bangladesh	CS (DHS)	1013 (induced abortion); 480 (unintended pregnancy)	Unwanted or mistimed pregnancy, and pregnancy termination	Serious
Kamal and Hassan [48]	2015	Bangladesh	CS (DHS)	15779	Unwanted or mistimed pregnancy, and pregnancy termination	Serious
Kidman [22]	2016	34 countries	CS (DHS)	39877	Intimate partner violence	Serious
Kidman and Heymann [23]	2018	47 countries in multiple regions	CS (DHS)	from 64100 to 338575	Number of timing of births; contraceptive use; intimate partner violence	Serious
Le Strat et al. [21]	2011	United States	CS	18645	Mental health	Serious
Misunas et al. [24]	2019	15 countries in multiple regions	CS (DHS)	not stated	Number and timing of births; Contraceptive use; others	Serious
Nasrullah et al. [49]	2013	Pakistan	CS (DHS)	1404	Maternal health care	Serious
Nasrullah, Muazzam, et al. [50]	2014	Pakistan	CS (DHS)	1560	Number of timing of births; unwanted or mistimed pregnancy, and pregnancy termination	Serious
Nasrullah, Zakar, et al. [51]	2014	Pakistan	CS (DHS)	589	Intimate partner violence	Serious
Nigatu et al. [52]	2018	Ethiopia	CS	322	Nutritional status	Serious
Olamijuwon et al. [53]	2017	18 countries in Sub-Saharan Africa	CS (DHS)	25327	Contraceptive use; intimate partner violence; maternal health care	Serious
Onagoruwa and Wodon [54]	2018	15 countries in South Asia and Sub-Saharan Africa	CS (DHS)	from 1441 to 10285	Number of timing of births	Serious
Oshiro et al. [55]	2011	Nepal	CS	905	Intimate partner violence	Serious
Pandey and Singh [56]	2015	India	CS (DHS)	54918	Contraceptive use	Serious
Paul [57]	2018	India	CS	35253	Unwanted or mistimed pregnancy, and pregnancy termination; others	Serious
Paul and Chouhan [58]	2019	India	CS (DHS)	190898	Maternal health care	Serious
Prakash et al. [59]	2011	India	CS (DHS)	39026	Others	Serious
Rahman et al. [60]	2014	Bangladesh	CS (DHS)	2174	Intimate partner violence	Serious
Rahman et al. [61]	2018	Bangladesh	Cohort	1183	Nutritional status	Serious
Raj [62]	2010	India	CS (DHS)	14628	Unwanted or mistimed pregnancy, and pregnancy termination; Birth intervals; contraceptive use; others	Serious
Raj et al. [63]	2009	India	CS (DHS)	14873	Number of timing of births; unwanted or mistimed pregnancy, and pregnancy termination; contraceptive use	Serious
Raj et al. [64]	2010	India	CS (DHS)	10514	Intimate partner violence	Serious
Raj et al. [65]	2013	Nepal	CS (DHS)	2439	Contraceptive use	Serious
Santhya et al. [66]	2010	India	CS	8314	Contraceptive use; unwanted or mistimed pregnancy, and pregnancy termination; maternal health care; intimate partner violence	Serious
Sekine and Carter [67]	2019	Nepal	CS (DHS)	3970	Maternal health care	Serious
Singh et al. [68]	2019	India	CS	350	Others	Critical
Solanke [69]	2019	Nigeria	CS (DHS)	25852	Number and timing of births	Serious
Speizer and Pearson [70]	2011	India	CS (DHS)	59841	Intimate partner violence	Serious
Tenkorang [71]	2019	Ghana	CS	2289	Intimate partner violence	Serious
Thakur et al. [72]	2015	India	Case-control	452	Others	Serious
Thekdi et al. [73]	2014	India	CS	400	Others	Critical
Uddin et al. [74]	2019	Bangladesh	CS (DHS)	16099	Maternal health care	Serious
Yaya et al. [75]	2019	34 countries in sub-Saharan Africa	CS (DHS)	60215	Number of timing of births; unwanted or mistimed pregnancy, and pregnancy termination; contraceptive use	Serious
Yimer et al. [76]	2016	Ethiopia	CS	434	Nutritional status	Serious
Yount et al. [77]	2016	Bangladesh	Cohort	3902	Intimate partner violence	Serious
Yusuf et al. [78]	2018	Nigeria	CS (DHS)	5277	Nutritional status	Serious

*Abbreviation: CS* cross-sectional

^a^Assessment differs depending on the outcome being assessed

### Bias assessment

All studies included in our review were determined to be at serious or critical risk of bias based on assessment using ROBINS-I. The summary risk of bias assessment for each study is listed in Table 1; risk of bias within each ROBINS-I domain in each study is detailed in Supplementary File 2. Confounding was the most prevalent concern. Every study was deemed to be at serious to critical risk of bias in this domain, most often because of failure to account for important sources of confounding and inappropriate adjustment for variables affected by age at marriage that are on the causal pathway. Cross-sectional surveys like the DHS often do not collect information necessary to control for confounding. Failure to control for major sources of confounding like childhood poverty and gender inequality may result in overestimation of the harmful effects of child marriage. The second common source of bias was adjustment for variables measured after marriage that are likely on the causal pathway between age at marriage and the health outcomes being studied. To illustrate, the authors of many studies included in this review acknowledged that age at marriage may dictate how long a girl stays in school and that her educational attainment may subsequently influence a wide range of health outcomes. Unfortunately, they then adjusted for educational attainment in regression analyses. This will very likely result in biased estimates because educational attainment was measured after marriage and is more likely to be a mediator than a confounder (Fig. 1) [79, 80]. Adjusting for it may remove some of the effect of child marriage on health and lead to underestimates of effect. Given that these two issues may bias results in different directions, predicting the net direction of confounding within studies is challenging. Other sources of bias also affected many of the studies in this review, including selection and measurement biases. Few authors discussed the potential influence of bias on their estimates or their conclusions.

### The health consequences of child marriage

Studies included in our review estimated the effect of child marriage on a variety of health outcomes. The most common outcomes were measures of reproductive health, such as fertility and fertility control, maternal health care utilization, intimate partner violence, mental health, and nutritional status. The following paragraphs synthesize the literature in each of these categories. In light of the serious risk of bias in all included studies, we interpreted these results with a high degree of caution. We assessed the direction of effect measures, meaning whether the study found that child marriage increased or decreased the probability of experiencing the outcome, and the consistency of directionality across studies within each outcome category. We also assessed the precision of effect measures by evaluating the width of confidence intervals surrounding those measures. We did not interpret the magnitude of the effect estimates from individual studies due to the risk of bias.

#### The effect of child marriage on the number and timing of births

Eleven studies estimated the effect of child marriage on the number of children born, though this outcome was not consistently measured. Some studies estimated the effect of child marriage on the odds of having given birth to any children [34, 50, 63], the odds of having three or more children [24, 46, 50, 63, 75], four or more children [34], five or more children [37, 69], or a continuous measure of the total number of children ever born [24, 25, 30, 46, 54]. The age ranges of the people included in these studies also differed, leading to variation in the time frame over which these births could have occurred. Child marriage was correlated with higher fertility in nearly all studies regardless of how the outcome was defined. The only exception was a study from Ethiopia that found no effect [30]. Ten of these studies focused on fertility exclusively among women. Misunas et al. [24] focused on men and came to similar conclusions: child marriage increased the odds that men aged 20-29 had fathered three or more children and increased the average number of children fathered by the ages of 40-49 [24].

A second commonly examined outcome was the likelihood of giving birth within the first year of marriage. Four studies based on data from South Asia [39, 46, 50, 63] and one study based on pooled data from multiple countries in Africa [75] examined this outcome. Three of these studies [46, 50, 75] reported that marriage before the age of 18 decreased the odds of giving birth within the first year of marriage. The remaining two [39, 63] did not find any evidence of a relationship between child marriage and this outcome.

We also identified five studies that estimated the effect of child marriage on the likelihood of giving birth before a specified age, often referred to as early, teen, or adolescent pregnancy [23, 26, 31, 32, 34]. Three of these studies found that child marriage increased the odds of giving birth before the age of 20 [26, 31, 32], the other two reported that child marriage increased the odds of giving birth before the age of 18 [23, 34]. Two studies also estimated the effect of child marriage on mean age at first birth and found that those who married before the age of 18 gave birth for the first time at younger ages, on average, than those who married at older ages [32, 46].

Collectively, this evidence indicates that women who marry as children often begin having children of their own at earlier ages when compared to their peers who marry after their 18th birthdays, and that they tend to have a larger number of children over their lifetimes. This is not surprising, given that marriage changes sexual behavior in ways that increase the risk of pregnancy. Essentially, girls who marry at earlier ages spend a longer time at risk of pregnancy than those who marry later.

#### The effect of child marriage on birth intervals

The World Health Organization recommends an interval of at least 24 months between a live birth and a subsequent pregnancy to reduce the risk of poor maternal health outcomes [81]. Five studies included in our review estimated the effect of child marriage on the likelihood of repeated childbirths in less than two years [39, 50, 62, 63, 75]. All five used samples of women between the ages of 20 and 24 who were included in DHS. A sixth study based on a small cross-sectional sample of women aged 15-49 from Ethiopia estimated the effect on repeated childbirth in less than three years [27]. These studies came to different conclusions. Two studies by the same author reported that child marriage increased the odds of repeated childbirth within two years in India [62, 63] but another study based on the same data source found that women who married as children were less likely to have two births within a two-year period than those who married at older ages [39]. There were also differences in the results of research from Pakistan: one study reported that child marriage made it more likely that women would have two births within two years [50] while another found no evidence that child marriage influenced this outcome [39]. Child marriage protected against short birth intervals in Nepal [39] and in an analysis of data from 34 African countries [75]. There was no evidence that child marriage influence the likelihood of short birth intervals in Bangladesh [39].

These results, which range from harmful to protective effects, indicate that child marriage is not clearly or consistently correlated with short birth intervals.

#### Child marriage, unwanted or mistimed pregnancy, and pregnancy termination

Seven studies estimated the effect of child marriage on the likelihood of experiencing a mistimed or unwanted pregnancy [39, 46, 47, 50, 62, 63, 75]. All seven were based on analyses of DHS data. The DHS typically asks women whether pregnancies were wanted at the time they occurred, wanted later (i.e., mistimed), or not wanted. Interestingly, six of the seven studies that examined this outcome reduced these categorical responses into a binary measure: women were categorized as having an unwanted pregnancy if they reported that they had a mistimed pregnancy *or* if they became pregnant when they did not want any more children [39, 46, 50, 62, 63, 75]. The rationale for doing this was not explained in any of the studies. The remaining study [47] only categorized instances in which a woman became pregnant at a time when she did not want any more children as unwanted.

Estimates of the effect of child marriage on this outcome are mixed. A study from 34 countries in Africa reported that child marriage protected against mistimed/unwanted pregnancies [75]. Studies from India, Pakistan, and Nepal concluded that child marriage increased the odds of experiencing mistimed/unwanted pregnancy [39, 50]. Three studies from Bangladesh came to different conclusions. One found no relationship between child marriage and this outcome [39] while another reported that child marriage increased the odds of mistimed/unwanted pregnancy [46]. The third used a different definition of the outcome and found that marriage before the age of 15 was positively associated with unwanted pregnancy (mistimed pregnancies were treated as wanted) but no evidence that marriage between the ages of 15 and 17 affected the likelihood of unwanted pregnancy [47].

Three of these studies also estimated the effect of child marriage on the likelihood of experiencing two or more mistimed or unwanted pregnancies [39, 62, 63]. Godha et al. reported a large effect of child marriage on having multiple mistimed/unwanted pregnancies in India, Bangladesh, and Pakistan but results were inconclusive in Nepal [39]. Two studies by the same author reported that child marriage increased the odds of having multiple mistimed/unwanted pregnancies in India [62, 63].

We identified eight studies of the effect of child marriage on pregnancy outcomes [39, 47, 48, 50, 57, 63, 66, 75]. Six of these relied on the DHS, which typically asks female respondents, “Have you ever had a pregnancy that miscarried, was aborted, or ended in a stillbirth?” [82]. The wording of this question makes it impossible to examine these outcomes separately. As a result, most studies based on the DHS used a composite outcome that grouped these three events despite differences in their intendedness. Five studies based on the DHS concluded that child marriage increased the odds of having a pregnancy end in either miscarriage, abortion, or stillbirth [39, 48, 50, 63, 75]. Exceptionally, the 2007 Bangladesh DHS asked a yes or no question regarding whether a woman had ever terminated a pregnancy. Using responses to this question, Kamal reported that marriage before the age of 15 was correlated with higher odds of termination but no evidence that marriage between 15 and 17 years of age influenced this outcome [47].

Two studies from India used other cross-sectional data sources and defined their outcomes differently. Santhya et al. used a combined outcome of miscarriage and stillbirth and found that child marriage increased the likelihood of experiencing either of these birth outcomes. [66]. Paul considered stillbirth and miscarriage separately. Marriage before the age of 15 increased the odds of stillbirth and miscarriage, but marriage between the ages of 15-17 was no less risky in this regard than marriage at 18 or later [57].

#### Child marriage and contraceptive use

Fifteen of the studies included in our review estimated the effect of child marriage on various aspects of contraceptive use [23, 24, 32, 39–41, 43, 46, 53, 56, 62, 63, 65, 66, 75]. All were based on cross-sectional data and thirteen used data from the DHS.

Of these fifteen studies, eight estimated the effect of child marriage on the likelihood that women were using contraception at the time the surveys were conducted [32, 39, 40, 46, 53, 62, 63, 65]. As with other outcomes, results were mixed. Child marriage reportedly increased the likelihood of using modern contraception in India and Bangladesh [39]. Results from Pakistan and Nepal indicate that the same may be true in those countries but the estimates were imprecise [39]. A second study from Nepal concluded that child marriage led to lower odds of using modern contraception [65]. The two studies from Nepal used different samples of women, which may partially explain the differences in their results. A study based on pooled data from 18 African countries found that child marriage was correlated with a lower likelihood of using modern contraception [53]. However, results varied markedly between countries and across geographic regions; in some, child marriage appeared to increase the likelihood of using modern contraception [53]. In Ghana, de Groot et al. found that child marriage was not correlated with the odds of using any form of contraception or with the use of modern contraceptives [32].

Two other studies investigated the effect of child marriage on the use of any method of contraception, including those not classified as modern [40, 46]. Marriage prior to the age of 15 led to lower odds of contraceptive use in Rwanda, but there was no indication that those who married between 15 and 17 years of age were any more or less likely to use contraception than those who married at older ages [40]. In Bangladesh, women who married as children were more likely to be using some form of contraception at the time of the survey than those who married at the age of 18 or older [46]. In yet another iteration of this outcome, Yaya [75] reported that women who married as children were more likely to have *ever* used modern contraception. A single study estimated the effect of child marriage among men on the likelihood that they were using modern contraception [24]. In five of ten countries studied, child marriage was not related to modern contraceptive use. In two (Honduras and Nepal), child marriage seemed to slightly increase the odds of contraceptive use, but it decreased the likelihood in Madagascar [24].

A second outcome that has received particular focus is whether a woman used contraception before her first pregnancy. All four studies that examined the effect of child marriage on this outcome were based on data from South Asia [39, 56, 63, 66] and concluded that marrying as a child decreased the likelihood that a woman used contraception prior to her first pregnancy [39, 56, 63, 66]. The authors of these studies frequently interpreted their results as an indicator of uncontrolled fertility that may place girls and their children at risk of poor health outcomes [39, 56, 63]. However, this relationship is more challenging to interpret because the outcome variables used did not capture whether pregnancies were desired shortly after marriage or the outcomes of those pregnancies.

Four studies estimated the impact of child marriage on the likelihood that a woman had an unmet need for contraception [23, 32, 41, 43]. This outcome was conceptually defined as a woman who is sexually active but not using contraception and who reports a desire to delay the next birth (a need for spacing), have no more births (a need for limiting), or a combination of the two. Once again, conclusions differ between studies. Using pooled DHS data from 47 countries, Kidman and Heymann found that marrying as a child increased the likelihood that women had an unmet need for contraception to either space or limit births [23]. An analysis of DHS data from Ethiopia found that women who married as children were less likely to have an unmet need for spacing and less likely to have an unmet need for limiting births compared to women who married at older ages [41]. In Zambia, child marriage was correlated with a greater unmet need for spacing and for limiting [43]. In Ghana, de Groot et al. found that child marriage was not correlated with an unmet need for limiting [32]. These studies all used different samples, which may partially explain the differences in their results.

#### Child marriage and use of maternal health care

Nine of the studies included in our review estimated the effect of child marriage on the use of health care during pregnancy, at the time of delivery, and during the post-partum period, which we collectively refer to as maternal health care [33, 39, 49, 53, 58, 62, 66, 67, 74].

Studies of prenatal care defined their outcomes as the receipt of at least one prenatal checkup [49, 62], the receipt of four or more prenatal checkups [49, 58, 67], or a count of the total number of prenatal checkups received [39, 53]. Once again, results within countries come to different conclusions. In Nepal, one study found that women who married as children were less likely to receive four or more prenatal checkups [67] while another found no evidence that child marriage influenced this outcome [39]. A study from India found no indication that child marriage affected prenatal care [39] but two others concluded that child marriage decreased the likelihood of receiving at least one checkup and of receiving at least four checkups [58, 62]. In one study from Pakistan, women who married as children were less likely to receive any prenatal care than those who married at older ages, but there was no difference in the likelihood of receiving four or more checkups [49]. A separate study from the same country reported that child marriage had no effect on the number of prenatal care checkups [39]. The effect of child marriage on the number of prenatal care visits varied between geographic regions in Africa. In some, child marriage appeared correlated with a decrease the number of visits while in others there was no effect [53].

Compared to other outcomes, the results of studies that estimated the impact of child marriage on the likelihood of delivering in a health care facility were remarkably consistent. Across geographic locations, all seven studies that examined this outcome concluded that child marriage reduced the likelihood of delivery in a health care facility [39, 49, 53, 58, 66, 67, 74]. Six of the same studies also found that women who married as children were less likely to have a skilled health care provider present during delivery [39, 49, 53, 58, 67, 74].

Only two studies considered post-natal care [58, 67]. One reported that child marriage led to lower likelihood of a post-natal checkup within 42 days of delivery in India [66] while the other found a lower likelihood of a checkup within 24 h of delivery in Nepal [75].

#### Child marriage and intimate partner violence

Sixteen studies estimated the effect of child marriage on the likelihood of experiencing intimate partner violence [22, 23, 29, 35, 38, 42, 51, 53, 55, 60, 62, 64, 66, 70, 71, 77]. Fifteen of these studies were based on cross-sectional data [22, 23, 29, 35, 38, 42, 51, 53, 55, 60, 62, 64, 66, 70, 71] and eight (50%) were based on the DHS [22, 23, 51, 53, 60, 62, 64, 70]. The DHS measures intimate partner violence by asking female respondents a series of questions regarding their experience of specific acts. For example, physical violence is assessed by asking women whether they have been slapped, kicked, or pushed, among other actions. Sexual violence is assessed by asking whether the respondent’s husband has forced her to have sex or perform sex acts when she did not want to. Emotional violence is measured by asking whether her spouse has humiliated or threatened her [83]. Studies based on data from sources other than the DHS tended to use the same or very similar questions to measure the experience of violence.

Physical violence was the most frequently examined outcome but was measured over different time frames across studies. Some estimated the likelihood of ever having experienced physical violence from a husband or partner while others considered only the year prior to the survey. Still, others focused on the 3 months prior to the survey [35], the 9 months between survey waves [77], or during pregnancy [38]. Regardless of the time period during which violence was measured, the conclusions of these studies were fairly consistent: nearly all reported that marrying as a child increased the likelihood of experiencing physical violence [22, 38, 51, 55, 60, 64, 66, 71, 77]. A study from Ethiopia found no indication that child marriage had an effect on this outcome but it considered a relatively short time period of 3 months [35].

Estimates of the effect of child marriage on the experience of sexual violence were much less consistent. Two studies from India came to conflicting conclusions. Raj et al. found that child marriage did not increase the likelihood of experiencing sexual violence at any point or in the year prior to the 2005-06 National Family Health Survey [64]. However, a study by Santhya et al. based on survey data collected from five Indian states between 2006 and 2008 found that child marriage did increase the likelihood of ever experiencing sexual violence [66]. Studies from Bangladesh and Ghana reported that women who married as children were no more or less likely to experience sexual violence than those who married at later ages [60, 71]. Two studies that pooled DHS data across multiple countries also found mixed results [22, 53]. Olamijuwon used data from 18 African countries and found that child marriage increased the odds of experiencing sexual violence in Central, East, and Southern Africa, but there was no evidence of a statistical relationship in West Africa [53]. Kidman used DHS data from 34 countries across the globe and reported that child marriage seemed to increase the odds of experiencing sexual violence in the year prior to the surveys in all included geographic regions except Europe and Central Asia [22]. Erulkar found that women who married as children in Ethiopia were more likely to report that their first sexual experience was forced [35].

Only two studies, one from Pakistan and one from Ghana, considered emotional violence as a stand-alone outcome. Both concluded the child marriage led to an increase in the likelihood of ever experiencing emotional violence from an intimate partner [51, 71].

Five studies considered only combined outcomes that mixed indicators of physical and sexual violence [62, 70], or physical, sexual, and emotional violence [23, 29, 42]. All of these found that child marriage was associated with increased reporting of these composite measures of violence, but some results were sensitive to the sample used and were inconsistent across locations [70]. Hong Le et al. considered whether child marriage affected the likelihood of violence among boys but was underpowered to detect any effect [42].

#### Child marriage and mental health

Five of the studies included in our review estimated the effect of child marriage on various aspects of mental health. These studies relied on cross-sectional data collected from Ghana, Iran, Ethiopia, Niger and the United States [21, 32, 36, 44, 45]. Women in the United States who married before the age of 18 were more likely to report experiencing a wide range of mood, anxiety, and other psychiatric disorders in adulthood when compared to those who married at later ages [21]. The authors of a small study from a single county in Iran found that women who married as children reported more depressive symptoms than those who married at the age of 18 or older [36]. John, Edmeades, and Murithi examined the relationship between child marriage and multiple domains of psychological well-being in Niger and Ethiopia [44]. The authors found that marriage before the age of 16 was correlated with poorer overall psychological well-being, but no evidence that marriage between the ages of 16 and 17 was associated with poorer outcomes when compared to women who married at the age of 18 or later [44]. In Ghana, child marriage seemed to protect against measures of stress. The Ghanaian study also found no indication of differences in levels of social support between women who married before the age of 18 and those who married after their 18th birthdays, though these odds ratio estimates were very imprecise [32].

#### Child marriage and nutritional status

Six studies included in our review estimated the effect of child marriage on indicators of nutritional status [28, 34, 52, 61, 76, 78]. Four focused exclusively on pregnant women. Two studies from Ethiopia examined the relationship between child marriage and mid-upper arm circumference (MUAC) [52, 76]. One reported that pregnant women who married before the age of 18 were more likely to have an MUAC less than 22 cm, often interpreted as a marker of undernutrition [84, 85], compared to those who married later on [52]. The other found that marrying before the age of 15 increased the likelihood of MUAC <22 cm but no evidence that marrying between the ages of 15 and 17 affected this outcome [76]. A third study from Ethiopia reported that child marriage led to an increase in the prevalence of Vitamin A deficiency among pregnant or recently post-partum women [28].

Two other studies focused on women who were not pregnant and used body mass index (BMI) as the indicator of nutritional status [34, 78]. Their results diverge. Yusuf et al. found that women in Nigeria who married as children were more likely to have a BMI less than 18.5, frequently interpreted as underweight among adults. However, in a study of 35 African countries, Efevbera et al. reported that child marriage was protective against being underweight (BMI<18.5) [44]. Interestingly, the authors of these studies offered plausible explanations for effects in either direction. Efevbera et al. hypothesize that girls who marry as children may gain access to more plentiful food at an earlier age and that repeated pregnancies during adolescence might result in greater weight gain relative to those who marry at later ages [34]. In contrast, Nigatu et al. note that repeat pregnancies in quick succession may have a detrimental impact on cumulative nutritional status [52]. This suggests that the mechanisms through which age at marriage may affect subsequent nutritional status have not been thoroughly considered.

#### Other health consequences of child marriage

A few of the studies included in our review examined outcomes other than those discussed above. We note them briefly here. A case-control study from India reported that women diagnosed with cervical cancer were more likely to have been married before the age of 18 [72]. A large, pooled analysis of DHS data from 47 countries reported that child marriage was associated with symptoms of sexually transmitted infections [23]. A small, cross-sectional study from a single Indian state found no evidence that child marriage led to an increase in the odds of obstetric fistula [68]. A third study from India examined the effect of child marriage on the odds of experiencing at least one complication during pregnancy, delivery, or within two months after delivery [57]. Marriage before the age of 15 seemed to increase the likelihood of pregnancy complications, but there was no evidence of an effect for marriage between 15 and 17 years. Child marriage was not associated with delivery complications, but was associated with postnatal complications [57]. A study from Ghana found no indication that child marriage influenced the likelihood of self-reported poor health, of being ill in the two weeks prior to the survey, or of having a health insurance card but did report that child marriage increased the odds of having difficulty with activities of daily living, such as bending or walking [32].

## Discussion

Our systematic review synthesized research on the health consequences of marrying before the age of 18. Studies almost uniformly found that women who married before the age of 18 began having children of their own at earlier ages and gave birth to more children over the course of their reproductive lives when compared to those who married at the age of 18 or later. Whether these outcomes, considered alone, are harmful to health is not clear. Though there are many reasons to be concerned about adolescent childbearing, none of the studies of the effect of child marriage on the timing of births considered whether those pregnancies were planned or desired or whether they resulted in obstetric complications or maternal morbidity or mortality [23, 26, 31, 32, 34, 39, 46, 50, 63, 75]. Similarly, having multiple births, especially at short intervals, may increase the risk of obstetric complications and subsequent morbidity or mortality. However, studies that compared the number of children born to women who married before the age of 18 with the number born to those who married at later ages also did not measure whether those pregnancies were planned or whether they led to harm [24, 25, 30, 34, 37, 46, 50, 54, 63, 69, 75]. Rather, studies seemed to assume that these are negative outcomes without directly measuring intentions or harms.

A separate set of studies that estimated the effect of child marriage on the experience of mistimed or unwanted pregnancies came to divergent conclusions: some found that child marriage increased the likelihood of these outcomes but others found that child marriage protected against them or had no effect. Studies of whether child marriage affected the likelihood of obstetric complications, miscarriage or stillbirth did not consider maternal age when those events occurred [39, 47, 48, 50, 57, 63, 66, 75]. Moreover, the fact that child marriage corresponds with a larger number of pregnancies means that girls who married prior to the age of 18 had more opportunities to experience these events compared to those who married later; this was not discussed in any of the studies we identified.

The results of studies in other outcome domains are very mixed and challenge some common narratives regarding child marriage. To illustrate, studies included in this review came to conflicting conclusions regarding whether child marriage increases or decreases the use of modern contraception, the likelihood of giving birth within the first year of marriage, and the likelihood of repeated childbirth within two years. Conclusions regarding mistimed and unwanted pregnancies were also mixed, as noted above. Collectively, these results suggest that child marriage is not uniformly characterized by an inability to control the number or timing of births and suggests that a more cautious approach to discussions of agency within these marriages is warranted, at least regarding fertility and fertility control.

Across studies, women who married as children were less likely to give birth in a health care facility or with assistance from a skilled health care provider. These findings raise concerns about access to emergency obstetric care and subsequent birth outcomes for both mother and child. However, we found only one study that estimated the effect of child marriage on the likelihood of complications during pregnancy, delivery, and the postpartum period [57] and consideration of the consequences for the infants born was beyond the scope of this review. This statistical relationship could be confounded by lack of access due to geographic distance. Child marriage is more common in rural areas, where health care facilities and skilled health care providers may be more spread out. It may also be a function of gender inequality, which may manifest as an inability to seek care without permission. Future research should consider the potential for confounding by these and other variables and investigate whether place modifies this relationship.

Child marriage could plausibly affect many aspects of maternal and reproductive health through complex causal pathways. However, most of the studies included in our review did not discuss causal mechanisms in detail, which may have hindered their ability to identify and account for various sources of bias. More thorough consideration and discussion of these mechanisms would strengthen the theoretical underpinnings of this body of literature and help mitigate biases. For example, use of Directed Acyclic Graphs to illustrate assumed causal relationships would help to clarify the causal pathways being studied and identify sources of bias [86].

The effects of child marriage among boys have been almost entirely overlooked. Only 2 of the 58 studies included in this review considered boys or men and one of them was underpowered to generate informative estimates [42]. This intense focus on child marriage among girls reflects the gendered nature of the practice. However, a substantial proportion of boys also marry before the age of 18 in some countries [7, 24] and further inquiry into the health consequences among boys is warranted.

The geographic distribution of research on child marriage and health is highly skewed. The focus on South Asia and sub-Saharan Africa may be justified since these regions have some of the highest rates of child marriage in the world. However, it is unclear why just three countries, India, Bangladesh, and Ethiopia, have received such focused attention while other countries in these regions have received very little. Child marriage is certainly ongoing in many other regions of the world that have received little or no research attention, including high-income countries [9, 87, 88].

The geographic distribution of these studies and the range of outcomes considered is clearly reflective of heavy reliance on the DHS. The DHS is appealing because it collects information on age at marriage that is comparable across settings and over time, data are readily accessible and of high quality, and samples are typically nationally representative. However, defaulting to this data source may also have restricted the range of outcomes studied. The DHS focuses primarily on reproductive health and our review included many studies of the effect of child marriage on fertility, contraceptive use, and intimate partner violence. Far less attention has been paid to other potential harms of child marriage that are not included in the surveys, such as indicators of mental health. Importantly, the DHS does not collect information on some of the strongest confounders of many relationships between child marriage and health, including childhood socioeconomic conditions and measures of gender equality. Other data sources will be necessary to increase the geographic scope of this body of research and to overcome some of the limitations inherent in the use of cross-sectional data to estimate causal effects.

All studies included in our review were at serious to critical risk of bias. Quantification of the net magnitude of different biases on the results of each study would have made the project untenable. Considering pervasive bias, we avoided interpreting the magnitude of reported estimates from individual studies and instead took only the directionality of the estimates at face value. This allowed us to assess the (in)consistency of conclusions within domains of health. However, it is entirely possible that bias could lead to a reversal of effects, i.e., estimating a positive effect when the true effect is negative or vice versa. The bias in these studies means that it is unclear whether any of the relationships described are causal.

Nearly all studies included in our review relied on cross-sectional data. There are severe limitations to using cross-sectional research designs to estimate causal effects, and more rigorous designs are needed to further our understanding of the consequences of child marriage. Quasi-experimental designs that more effectively mitigate confounding would strengthen this body of literature and have already been used to study the effect of child marriage on educational attainment and literacy. For example, Field and Ambrus and Sunder used age at menarche as an instrumental variable to study the effect of child marriage on these outcomes [3, 4]. Encouragement trials that randomly assign exposure to interventions meant to prevent child marriage could also be used to estimate the effects of child marriage on health outcomes, though such trials are more resource intensive to conduct [89]. However, given that the DHS and other cross-sectional data sources will likely continue to be used to investigate these relationships, the use of quantitative bias analyses to examine how sensitive estimates are to various sources of bias would be an improvement [90].

There are several limitations to this systematic review. First, to capture as wide a range of health outcomes as possible, we searched databases focused on human health and biomedicine. Relevant studies from other academic disciplines such as economics and sociology may have been missed using this approach. Second, our search was conducted in English and all included studies were published in English. Eligible studies published in other languages may have been missed, which could influence our conclusions regarding the geographic distribution of research. Finally, as noted in the introduction, child marriage may have consequences beyond the domain of health. We focused our systematic review on the health consequences of child marriage in response to growing rhetoric regarding child marriage as a population health concern. Rigorous systematic reviews of the effect of child marriage on educational and economic outcomes would be a valuable addition to the literature.

## Supplementary Information

**Additional file 1.**

**Additional file 2.**

**Additional file 3.**

## Data Availability

The PROSPERO protocol and the data extraction form are publicly available through the Open Science Foundation at https://osf.io/32mu7/.

## Abbreviations

BMI: Body Mass Index
CS: Cross-Sectional
DAG: Directed Acyclic Graph
DHS: Demographic and Health Surveys
MUAC: Mid-Upper Arm Circumference
ROBINS-I: Risk Of Bias In Non-randomised Studies - of Interventions tool
SES: Socio-Economic Status
UNFPA: United Nations Population Fund
UNICEF: United Nations Children’s Fund
