# Supplementary material for: The health consequences of child marriage: a systematic review of the evidence
Source: BMC Public Health. 2022 Feb 14;22:309. doi: 10.1186/s12889-022-12707-x (PMC8845223; doi:10.1186/s12889-022-12707-x)
Supplement: Supplementary file 1 — Additional file 1. [file 12889_2022_12707_MOESM1_ESM.docx]

**Supplementary File 1**

Search terms used in MEDLINE, Embase, and Ovid Global Health are as follows:

((early adj2 marriage*) or "minimum age for marriage*" or ((adolescen* or child* or teen* or "under age*" or underage* or "under 18" or "below the age of 18" or young or youth) adj (bride* or groom* or husband* or marriage* or married or nuptial* or wife or wives)) or ((adolescen* or child* or teen* or "under age*" or underage* or "under 18" or "below the age of 18" or young or youth) adj5 ("arranged marriage*" or "forced marriage*" or "servile marriage*" or "age at first marriage"))).mp.

Search terms using in Google Scholar:

intitle:"child bride"|intitle:"child marriage"|intitle:"adolescent marriage"|intitle:"early marriage"|intitle:"teen marriage" health
