# Supplementary material for: The health consequences of child marriage: a systematic review of the evidence
Source: BMC Public Health. 2022 Feb 14;22:309. doi: 10.1186/s12889-022-12707-x (PMC8845223; doi:10.1186/s12889-022-12707-x)
Supplement: Supplementary file 2 — Additional file 2. [file 12889_2022_12707_MOESM2_ESM.docx]

**Supplementary File 2 Risk of bias assessments of included studies using ROBINS-I**

| Author | Year | Confounding | Selection bias | Measurement of intervention | Bias due to deviation from intended intervention | Bias due to missing data | Measurement of outcome | Selective Reporting | Overall |
| --- | --- | --- | --- | --- | --- | --- | --- | --- | --- |
| Agyei and Mbamanya | 1989 | Serious | Low | Moderate | Low | NI | Low | Moderate | Serious |
| Ali et al. | 2020 | Serious | Low | Moderate | Low | Low | Low | Moderate | Serious |
| Ayane et al. | 2019 | Serious | Low | Moderate | Moderate | Low | Moderate | Moderate | Serious |
| Baytekus et al. | 2019 | Serious | Serious | Serious | Low | Low | Low | Moderate | Serious |
| Begum et al. | 2015 | Serious | Serious | Moderate | Moderate | Low | Moderate | Serious | Serious |
| Berlie and Alamerew | 2018 | Serious | Low | Moderate | Low | NI | Low | Moderate | Serious |
| Birhanu et al. | 2019 | Serious | Critical | Moderate | Low | Low | Moderate | Moderate | Critical |
| De Groot et al. | 2018 | Serious | Serious-Critical^*^ | Moderate | Low | NI | Moderate | Moderate | Serious-Critical^*^ |
| Delprato and Akyeampong | 2017 | Serious | Moderate | Moderate | Low | Low | Low | Moderate | Serious |
| Efevbera et al. | 2019 | Serious | Serious | Moderate | Moderate | Low | Moderate | Moderate | Serious |
| Erulkar | 2013 | Serious | Low | Moderate | Low | Serious | Moderate | Moderate | Serious |
| Fakhari et al. | 2020 | Serious | Low | Serious | Low | NI | Low | Moderate | Serious |
| Gebremedhin and Betre | 2009 | Serious | Low | Moderate | Low | Low | Low | Moderate | Serious |
| Gebrezgi et al. | 2017 | Serious | Low | Moderate | Low | Low | Moderate | Serious | Serious |
| Godha et al. | 2013 | Serious | Low | Moderate | Low | Low | Moderate | Moderate | Serious |
| Habyarimana and Ramroop | 2018 | Serious | Low | Moderate | Moderate | Low | Moderate | Moderate | Serious |
| Hailemariam and Haddis | 2011 | Serious | Low | Moderate | Moderate | Low | Moderate | Moderate | Serious |
| Hong Le et al. | 2014 | Serious - Critical^*^ | Low | Moderate | Low | Low | Moderate | Moderate | Serious |
| Imasiku et al. | 2013 | Serious - Critical^*^ | Low | Moderate | Low | Low | Moderate | Moderate | Serious |
| John, Edmeades, & Murithi | 2019 | Serious | Moderate | Moderate | Moderate | Serious | Low | Moderate | Serious |
| John, Edmeades, Murithi, et al. | 2019 | Serious | Serious | Moderate | Moderate | Serious | Low | Moderate | Serious |
| Kamal | 2012 | Serious | Low | Moderate | Moderate | Moderate | Low | Moderate | Serious |
| Kamal | 2013 | Serious | Low | Moderate | Moderate | Low | Moderate | Moderate | Serious |
| Kamal and Hassan | 2013 | Serious | Low | Moderate | Low | NI | Moderate | Moderate | Serious |
| Kidman | 2017 | Serious | Low | Moderate | Low | Low | Moderate | Moderate | Serious |
| Kidman and Heymann | 2018 | Serious | Moderate | Moderate | Moderate | Low | Moderate | Moderate | Serious |
| Le Strat et al. | 2011 | Serious | Serious | Moderate | Low | Moderate | Low | Moderate | Serious |
| Misunas et al. | 2019 | Serious | Low | Moderate | Moderate | NI | Moderate | Moderate | Serious |
| Nasrullah, Muazzam, et al. | 2014 | Serious | Low | Moderate | Low | Moderate | Moderate | Moderate | Serious |
| Nasrullah et al. | 2013 | Serious | Serious | Moderate | Low | Low | Moderate | Moderate | Serious |
| Nasrullah, Zakar, et al. | 2014 | Serious | Serious | Moderate | Low | NI | Moderate | Moderate | Serious |
| Nigatu et al. | 2018 | Serious | Low | Moderate | Low | Moderate | Low | Moderate | Serious |
| Olamijuwon et al. | 2017 | Serious | Serious | Moderate | Low | Serious | Moderate | Moderate | Serious |
| Onagoruwa and Wodon | 2018 | Serious | Low | Moderate | Moderate | Moderate | Low | Moderate | Serious |
| Oshiro et al. | 2011 | Serious | Serious | Moderate | Moderate | NI | Moderate | Serious | Serious |
| Pandey and Singh | 2015 | Serious | Low | Moderate | Low | NI | Moderate | Moderate | Serious |
| Paul | 2018 | Serious | Low | Moderate | Moderate | NI | Moderate | Moderate | Serious |
| Paul and Chouhan | 2019 | Serious | Low | Moderate | Moderate | NI | Moderate | Moderate | Serious |
| Prakash et al. | 2011 | Serious | Serious | Moderate | Moderate | NI | Moderate | Moderate | Serious |
| Rahman et al. | 2014 | Serious | Low | Moderate | Low | NI | Moderate | Moderate | Serious |
| Rahman et al. | 2018 | Serious | Low | Moderate | Low | Low | Low | Moderate | Serious |
| Raj | 2010 | Serious | Low | Moderate | Low | NI | Moderate | Moderate | Serious |
| Raj et al. | 2009 | Serious | Serious | Moderate | Low | NI | Moderate | Serious | Serious |
| Raj et al. | 2010 | Serious | Low | Moderate | Low | Low | Moderate | Moderate | Serious |
| Raj et al. | 2013 | Serious | Low | Moderate | Low | NI | Moderate | Serious | Serious |
| Santhya et al. | 2010 | Serious | Serious | Moderate | Low | NI | Moderate | Moderate | Serious |
| Sekine and Carter | 2019 | Serious | Serious | Moderate | Moderate | Low | Moderate | Moderate | Serious |
| Singh et al. | 2019 | Serious | Critical | Moderate | Moderate | NI | Moderate | Critical | Critical |
| Solanke | 2019 | Serious | Serious | Moderate | Moderate | NI | Moderate | Moderate | Serious |
| Speizer and Pearson | 2011 | Serious | Serious | Moderate | Low | NI | Moderate | Serious | Serious |
| Tenkorang | 2019 | Serious | Low | Moderate | Moderate | NI | Moderate | Moderate | Serious |
| Thakur et al. | 2015 | Serious | Serious | Moderate | Low | Low | Low | Serious | Serious |
| Thekdi et al. | 2014 | Critical | Low | Serious | Moderate | NI | NI | Serious | Critical |
| Uddin et al. | 2019 | Serious | Low | Moderate | Low | NI | Moderate | Serious | Serious |
| Yaya et al. | 2019 | Serious | Serious | Moderate | Low | NI | Moderate | Moderate | Serious |
| Yimer et al. | 2016 | Serious | Low | Moderate | Low | Low | Low | Moderate | Serious |
| Yount et al. | 2016 | Serious | Serious | Moderate | Low | NI | Moderate | Moderate | Serious |
| Yusuf et al. | 2018 | Serious | Serious | Moderate | Moderate | NI | Low | Moderate | Serious |

NI: No information.

* Assessment differs depending on the outcome being assessed.
